# Supplementary material for: State formation across cultures and the role of grain, intensive agriculture, taxation and writing
Source: Nat Hum Behav. 2025 Nov 25;10(1):156–63. doi: 10.1038/s41562-025-02365-5 (PMC12846917; doi:10.1038/s41562-025-02365-5)
Supplement: Supplementary file 1 — Supplementary Tables 1–6, Figs. 1–10 and Discussion. [file 41562_2025_2365_MOESM1_ESM.pdf]

nature human behaviour

Supplementary information https://doi.org/10.1038/s41562-025-02365-5

State formation across cultures and the role of grain, intensive agriculture, taxation and writing

In the format provided by the authors and unedited

Supplementary Information

Data

The data used in this study are taken from the Ethnographic Atlas (EA) (1), and the Standard Cross Cultural Sample (SCCS) (2), a subset of the EA. The societies in the EA and SCCS were matched to the global phylogeny (3). The language used by each EA society was checked using Glottolog (https://glottolog.org/). Only if the language name on the phylogeny is the same as the society name or Glottolog clearly identifies that the language is spoken by that society is the society included. This is a conservative approach that ensures that the EA and SCCS societies used are accurately matched to the phylogeny.

Table S1 Description and coding of variables.

**EA and SCCS Variable**	**Full Coding**	**Binary Traits**		
		**Binary Coding**	**No. of EA Societies**	**No. of SCCS Societies**
**EA028 & SCCS232 Agriculture: intensity**	1. None<br>2. Casual agriculture, incidental to other subsistence modes<br>3. Extensive or shifting agriculture, long fallow, and new fields cleared annually<br>4. Horticulture, vegetal gardens, or groves of fruit trees<br>5. Intensive agriculture, using fertilization, crop rotation, or other techniques to shorten or eliminate fallow period<br>6. Intensive irrigated agriculture	0 = 1 – 4 (Intensive Agriculture – absent)<br>1 = 5 & 6 (Intensive Agriculture – present)	626<br><br><br><br>241	-<br><br><br><br>-

**EA033 & SCCS237 Jurisdictional hierarchy beyond local community**	1. Acepholous - No levels<br>2. One level (e.g., petty chiefdoms)<br>3. Two levels (e.g., larger chiefdoms)<br>4. Three levels (e.g., states)<br>5. Four levels (e.g., large states)	0 = 1 – 3 (State absent)<br>1 = 4 & 5 (State present)	750<br><br>92	147<br><br>27
**EA029 & SCCS233 Agriculture: major crop type**	1. No Agriculture<br>2. Non-food crops only<br>3. Vegetables<br>4. Tree fruits<br>5. Roots or tubers<br>6. Cereal grains	0 = 1 – 5 (Grains absent)<br>1 = 6 (Grains present)<br><br>0 = 1,2 & 6 (Non-grain agriculture - absent)<br>1 = 3,4 & 5 (Non-grain agriculture - present)	379<br><br>483<br><br>483<br><br>379	90<br><br>84<br><br>-<br><br>-
**SCCS784 (No) Taxation Paid to Community (4)**	1. Regular Taxes<br>2. Only in special cases, modest<br>3. None	0 = 2 & 3 (Tax absent)<br>1 = 1 (Tax present)	-<br><br>-	53<br><br>30
**SCCS149 Writing and Records (5)**	1. None<br>2. Mnemonic devices<br>3. Nonwritten records<br>4. True writing; no records*<br>5. True writing, records	0 = 1 – 3 (Writing absent)<br>1 = 4 & 5 (Writing present)**	-<br><br>-	138<br><br>38

*The society has an indigenous system of writing but lacks any significant accumulation of written records, or alternatively has long used the script of alien people. **(Following 6, 7)

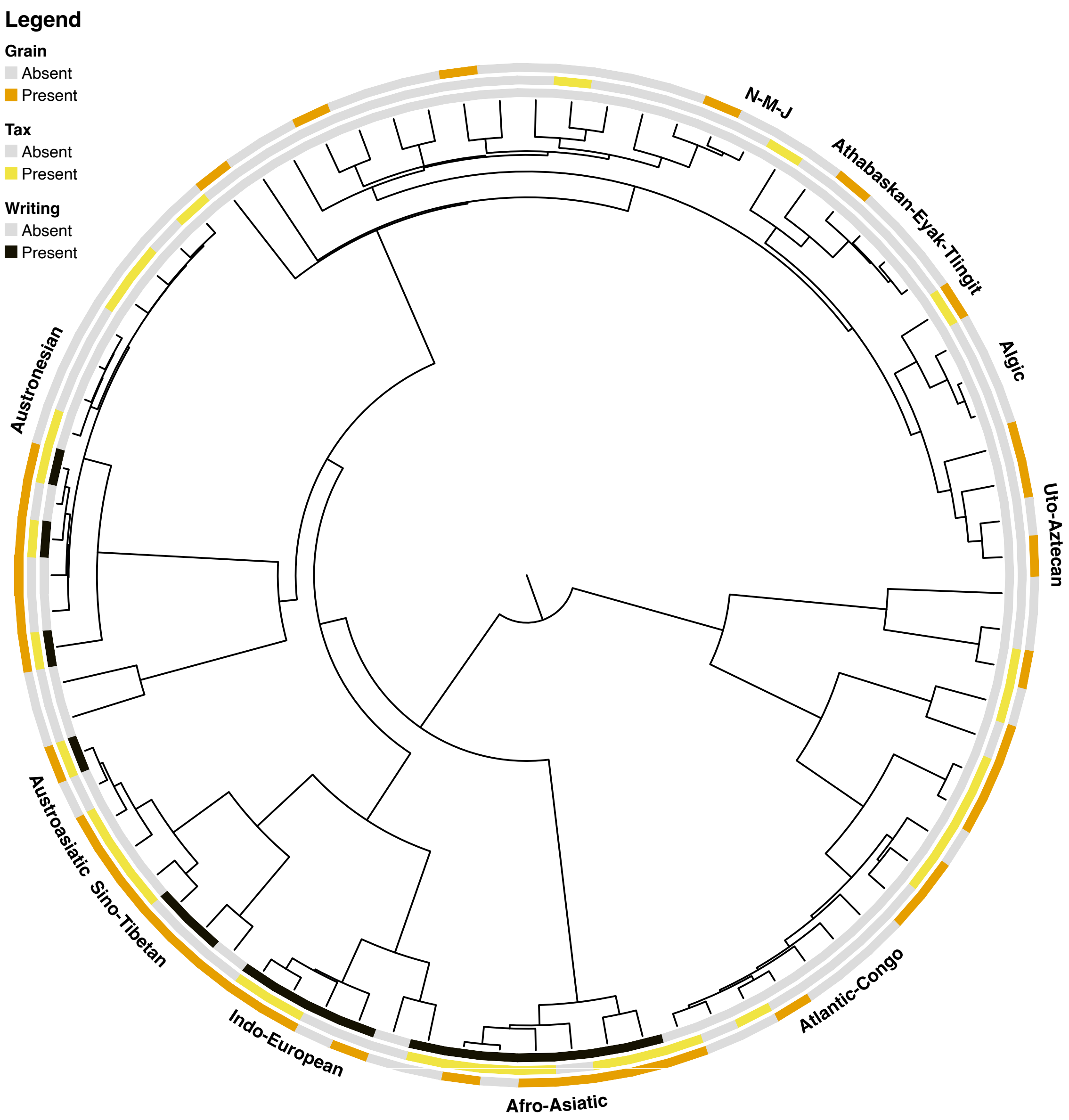

Figure S1 Phylogenetic distribution of data for Grain, Taxation and Writing. Maximum Clade Credibility Tree (MCCT) of the global treeset (3) with large language families highlighted, matched to the data for grain, taxation and writing from the Standard Cross-Cultural Sample (SCCS). (produced using Treeviewer (8)).

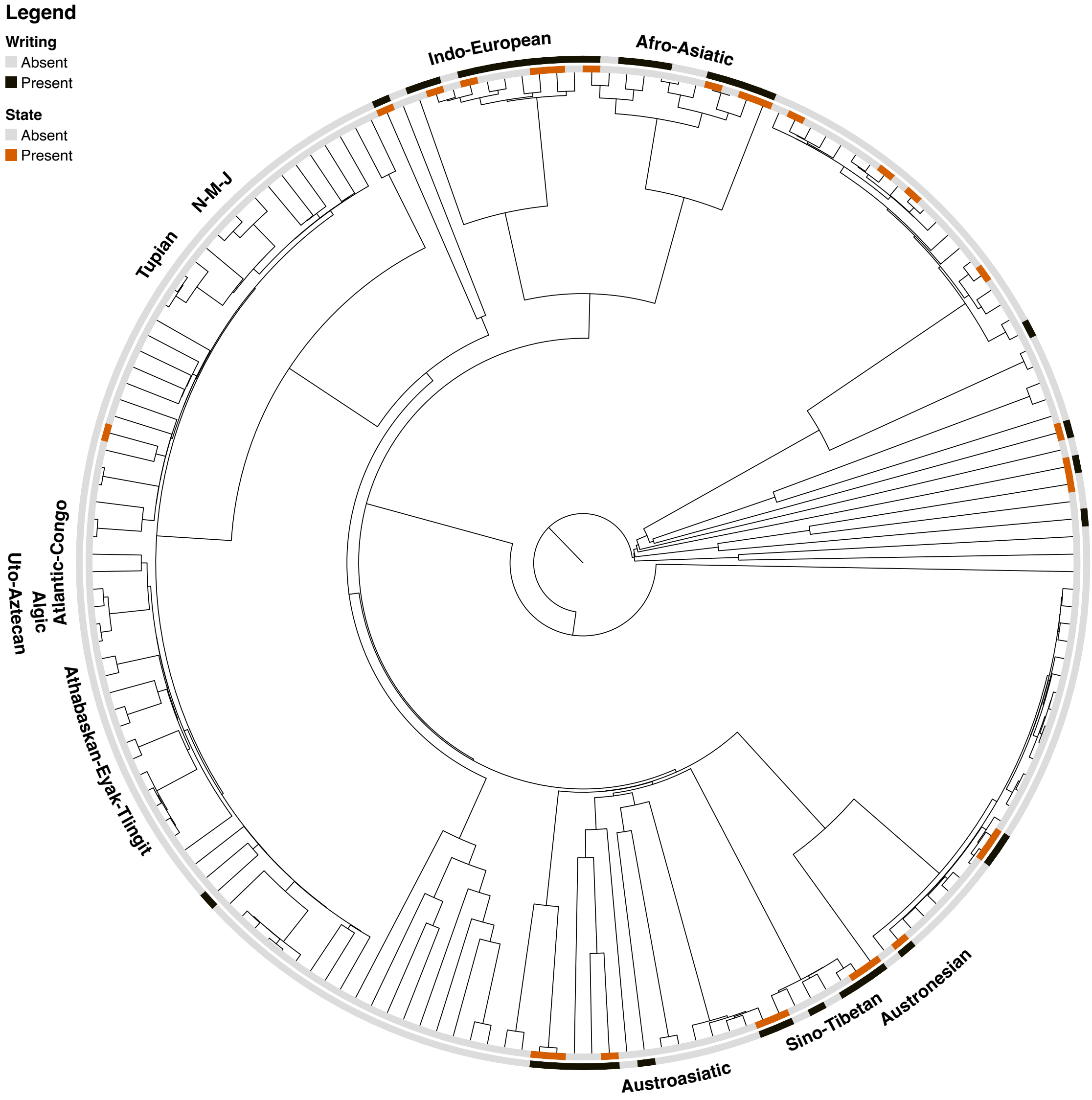

Figure S2 Phylogenetic distribution of data for State and Writing. Maximum Clade Credibility Tree (MCCT) of the global treeset (3) with large language families highlighted, matched to the data for state and writing from the Standard Cross-Cultural Sample (SCCS). (produced using Treeviewer (8)).

Geographic Location of Societies

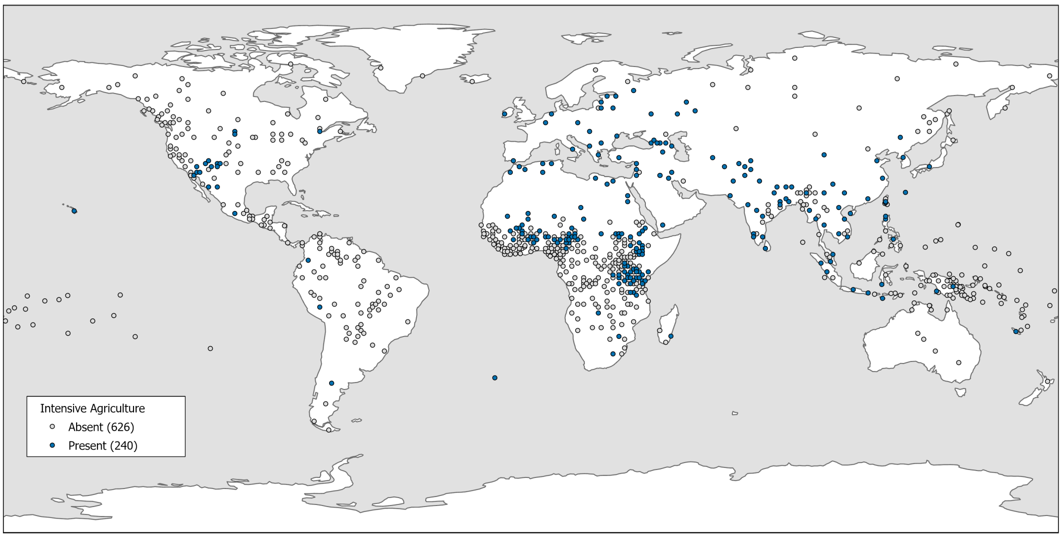

Figure S3. Geographic distribution of Intensive Agriculture. Plot of EA data for societies with intensive agriculture. The base map for this Figure was obtained from ESRI's World Terrain Reference (https://goto.arcgisonline.com/maps/Reference/World_Reference_Overlay) and generated using ArcGIS Pro 3.5.3. (9)

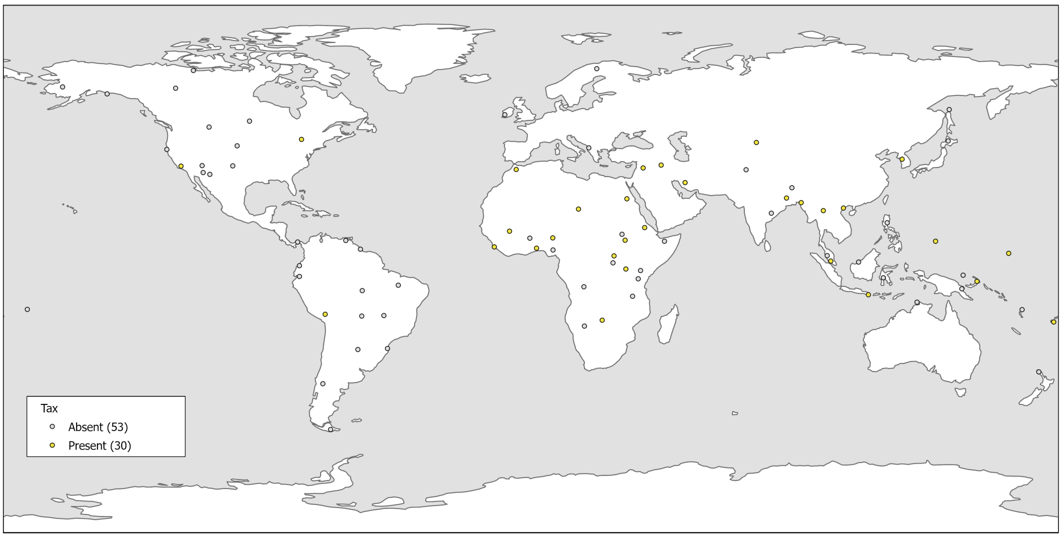

Figure S4. Geographic distribution of Taxation. Plot of SCCS data on societies that raise taxes. The base map for this Figure was obtained from ESRI's World Terrain Reference (https://goto.arcgisonline.com/maps/Reference/World_Reference_Overlay) and generated using ArcGIS Pro 3.5.3.(9)

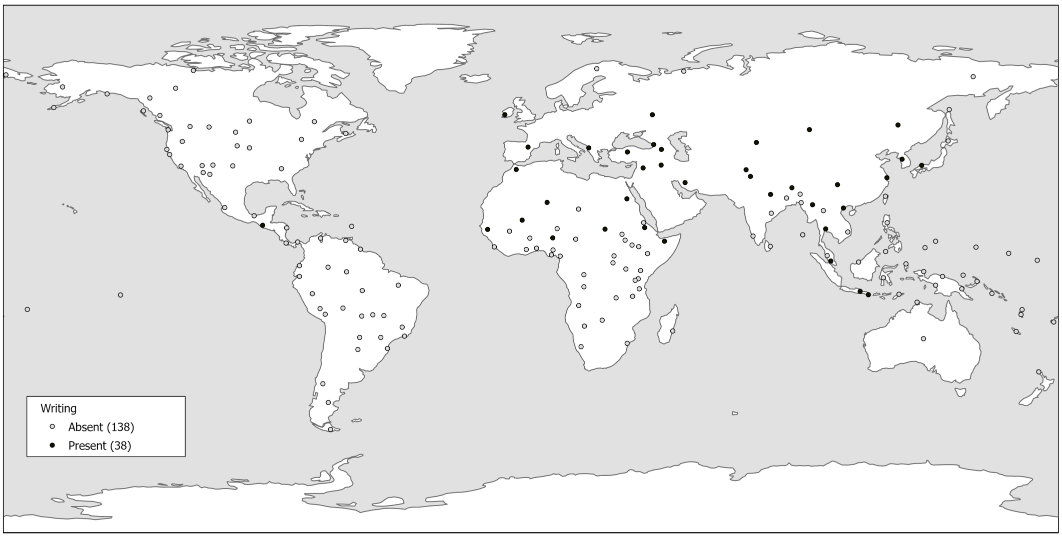

Figure S5. Geographic distribution of Writing. Plot of SCCS data on societies with writing. The base map for this Figure was obtained from ESRI's World Terrain Reference (https://goto.arcgisonline.com/maps/Reference/World_Reference_Overlay) and generated using ArcGIS Pro 3.5.3.(9)

Phylogenetic Signal

The phylogenetic signal, D (10), ranges from 0.28 to 0.78, and is significantly different from both random and a pure Brownian Motion expectation (Table S2) for all variables (except Writing, Brownian Motion p=0.10) consistent with models in which some but not all of the variation in each trait is explained by evolution along the branches of the phylogenetic tree.

Table S2. Phylogenetic signal of variables (D) (10). Random and Brownian motion - significance tests for model of evolution of variable. Estimates from 1,000 permutations.

SCCS Variable	D	Random	Brownian Motion
**SCCS232 Agriculture: intensity**	0.78	0.02	0.00
**SCCS237 Jurisdictional hierarchy beyond local community**	0.66	0.00	0.00
**SCCS233 Agriculture: major crop type**	0.43	0.00	0.00
**SCCS784 (No) Taxation Paid to Community (4)**	0.73	0.03	0.00
**SCCS149 Writing and Records (5)**	0.28	0.00	0.10

Correlated Evolution

For the analysis of correlated evolution between trait pairs we used the Discrete Method in BayesTraits (11). The likelihood of the dependent model, where traits evolve together across the phylogeny, is compared to the independent model, where traits are prevented from evolving together. We used the Stepping-Stone Sampler method (12) using 100 stones for 10,000 iterations to compare the log marginal likelihood of the dependent and independent models for each pair of traits (Table S3). Log Bayes Factor values were calculated such that <2 shows weak evidence, >2 positive evidence, 5-10 strong evidence, and >10 very strong evidence (13).

Table S3. Likelihoods for dependent and independent models of correlated evolution between traits across the global language phylogeny (3). The log Bayes factor shows the relative support for the dependent over the independent model. Log Bayes Factors = 2(log marginal likelihood complex model – log marginal likelihood simple model). Value <2 weak evidence, >2 positive evidence, 5-10 strong evidence, >10 very strong evidence (13).

Traits	Dependent model (log marginal likelihood)	Independent model (log marginal likelihood)	Log Bayes Factor
Intensive Agriculture/State emergence	- 660.23	- 687.02	53.56
Grain Agriculture/State emergence	- 692.63	- 703.93	22.59
Non-grain Agriculture/ State emergence	- 645.09	- 648.85	7.51
Grain Agriculture/Taxation	- 112.28	- 113.97	3.38
Taxation/Writing	- 125.41	- 135.36	19.90
Writing/State emergence	- 136.16	- 160.36	48.40

Atlantic-Congo Language Family

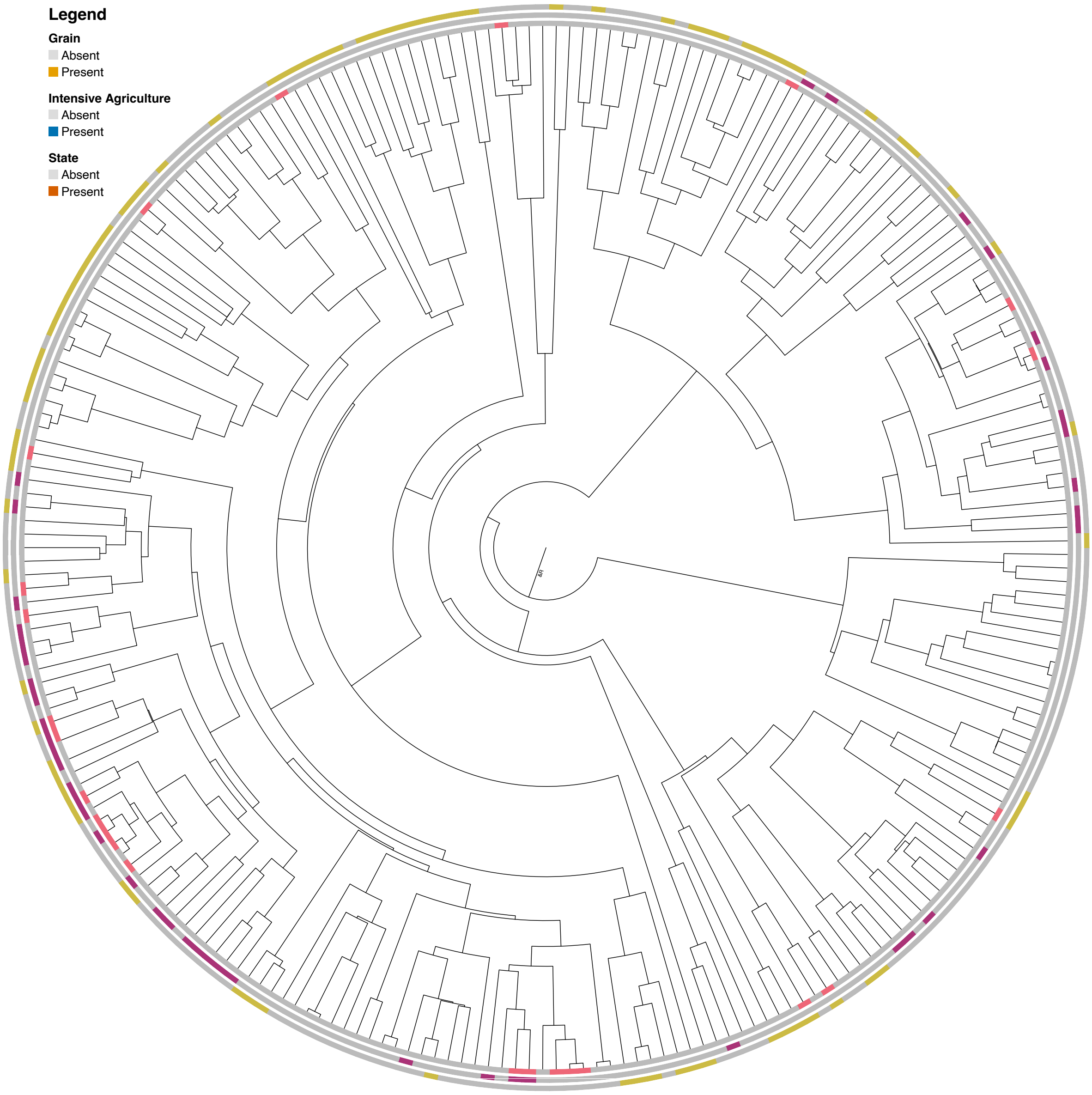

Figure S6. Phylogenetic distribution of data for State, Intensive Agriculture and Grain for the Atlantic-Congo language family. Maximum Clade Credibility Tree (MCCT) of the Atlantic-Congo treeset matched to the data for grain, intensive agriculture and state from the Ethnographic Atlas (EA). (produced using Treeviewer (8)).

Table S4. Likelihoods for dependent and independent models of correlated evolution between traits in the Atlantic-Congo language tree. The log Bayes factor shows the relative support for the dependent over the independent model. Log Bayes Factors = 2(log marginal likelihood complex model – log marginal likelihood simple model). Value <2 weak evidence, >2 positive evidence, 5-10 strong evidence, >10 very strong evidence (13).

Traits	Dependent model (log marginal likelihood)	Independent model (log marginal likelihood)	Log Bayes Factor
Intensive Agriculture/State emergence	-188.84	-186.17	-
Grain Agriculture/State emergence	-223.46	-220.50	-
Non-grain Agriculture/ State emergence	-223.23	-220.41	-

Rate Matrices

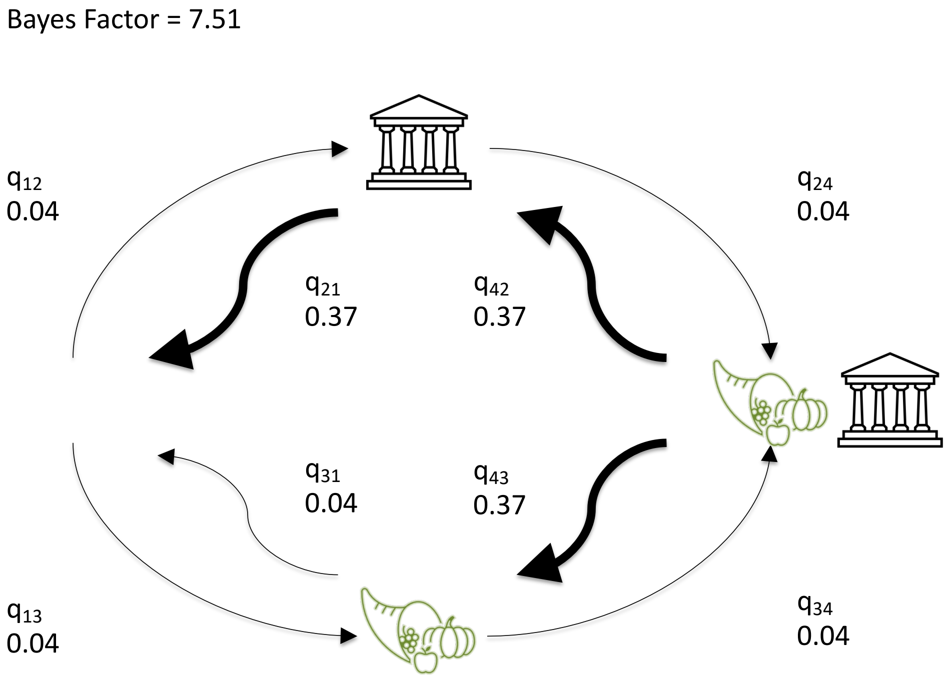

Figure S7 Correlated evolution between non-grain agriculture and the emergence of states. Strong correlated evolution (BF = 7.51), with a low q31 rate, but a high q42 rate, suggesting that the loss of non-grain agriculture was much more likely in states than in non-states. Non-grain agriculture: vegetables, tree fruits, roots, and tubers. The width of each arrow is equivalent to the rate of change between the state of the traits.

Bayes Factor = 3.38

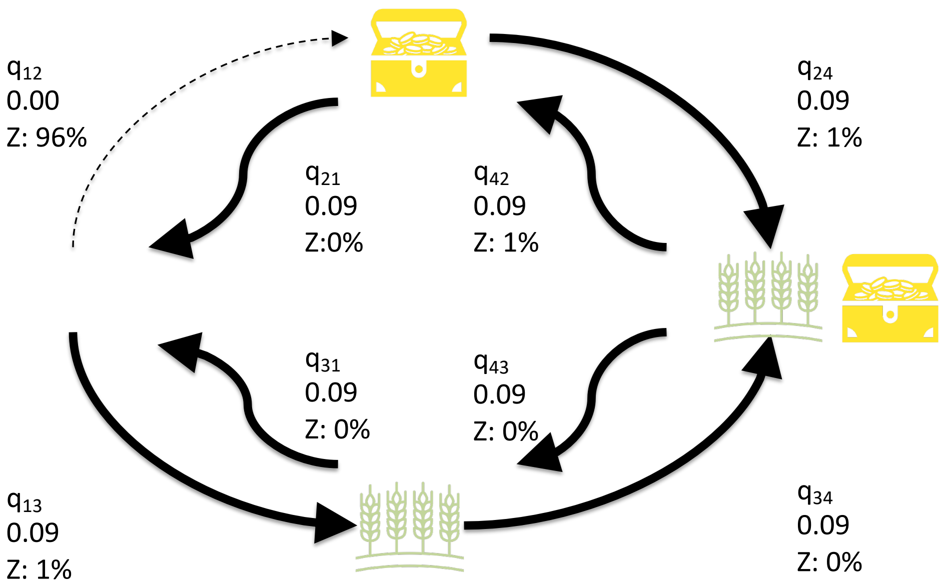

Figure S8 Correlated evolution between grain agriculture and taxation. Positive evidence of correlated evolution (BF = 3.38), with a high q34 rate and a zero q12 rate suggesting the shift to grain agriculture consistently predicts taxation. The width of each arrow is equivalent to the rate of change between the state of the traits.

Bayes Factor = 19.90

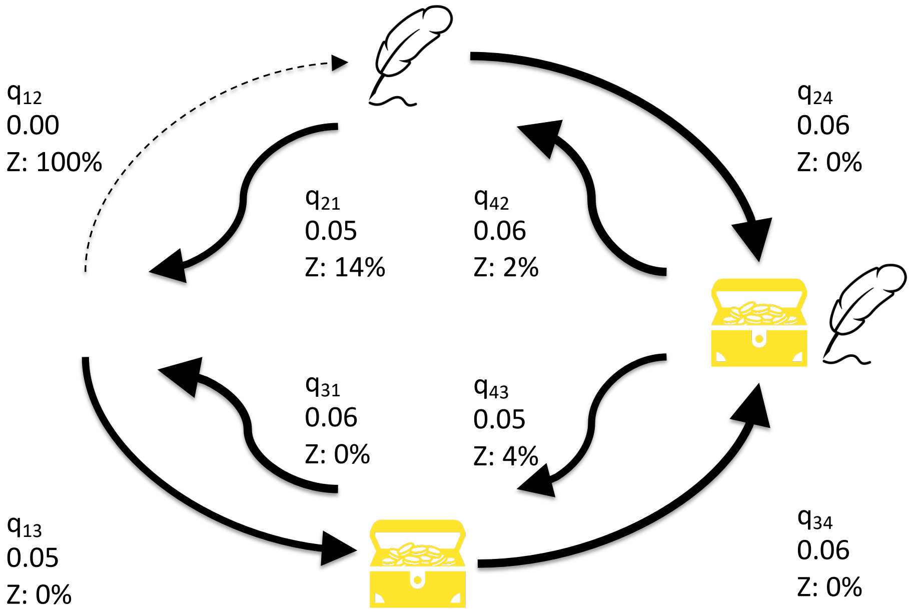

Figure S9 Correlated evolution between taxation and the adoption of writing. Very strong correlated evolution (BF = 19.90), with a very high rate for the adoption of writing with taxation (q34) and a zero rate without (q12), suggesting the shift to taxation consistently predicts the adoption of writing. The width of each arrow is equivalent to the rate of change between the state of the traits.

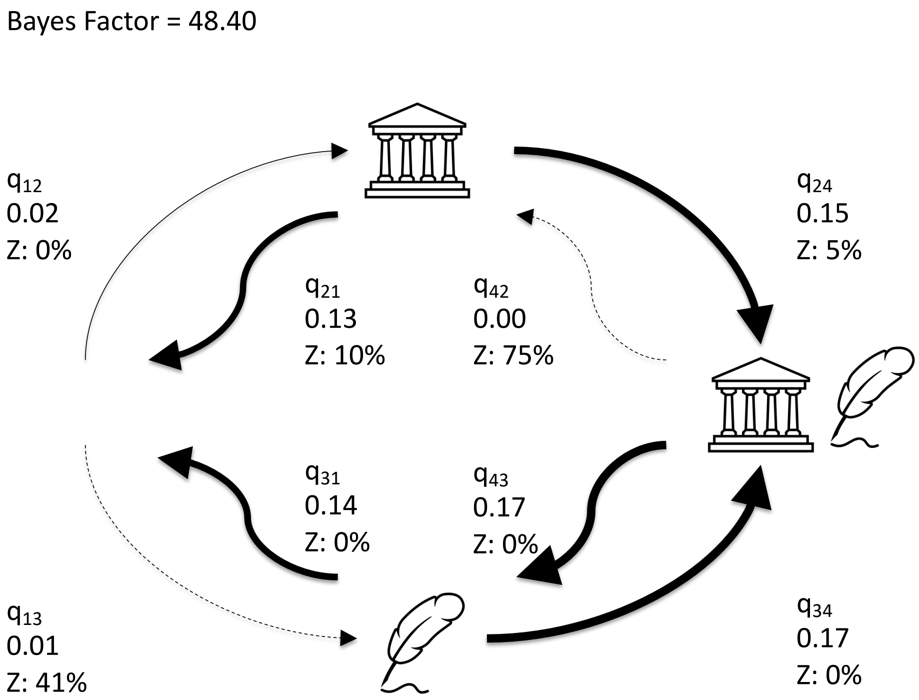

Figure S10 Correlated evolution between the adoption of writing and the emergence of states. Very strong correlated evolution (BF = 48.40), with low but non-zero rates for q12 and q13 suggesting the adoption of writing coevolves with the emergence of states and a zero rate for q42 suggesting that states do not lose writing once adopted. The width of each arrow is equivalent to the rate of change between the state of the traits.

Additional Robustness Checks

In order to evaluate the robustness of our inferences to model misspecification related to more recent horizontal transmission, we reran the analyses constraining all traits to be absent at the ancestral node of each of the 10 major language families. With the analyses including grain, we then allowed grain to be unconstrained, while the other trait was constrained to absent. We report the support for correlated evolution in these analyses (Table S5) and the transition rates between states compared to the unconstrained analyses (Table S6).

Table S5. Correlated evolution between binary traits with trait values at language family roots constrained compared to the unconstrained model. Log Bayes Factors: <2 weak evidence, >2 positive evidence, 5-10 strong evidence, >10 very strong evidence (13).

Traits	Unconstrained (from Table S3)	Both traits set as absent*	Grain unconstrained - other trait absent*
Intensive Agriculture/State emergence	53.56	61.85	_
Grain Agriculture/State emergence	22.59	32.24	23.73
Non-Grain Agriculture/State emergence	7.51	7.49	_
Grain Agriculture/Taxation	3.38	2.79	1.41
Taxation/Writing	19.90	9.15	_
Writing/State emergence	48.40	43.88	_

* At the ancestral node of the 10 largest global language families.

Transition Rates

Table S6. Transition rates from analysis of binary traits with trait values at language family roots constrained compared to the unconstrained model in reported results.

Variables	Constraint	Transition Rates							
		q12	q13	q21	q24	q31	q34	q42	q43
Intensive Agriculture/State emergence	None	0.03	0.05	0.31	0.31	0.27	0.06	0.04	0.31
	Absent*	0.04	0.05	0.39	0.40	0.34	0.07	0.03	0.39
Grain Agriculture/State emergence	None	0.00	0.07	0.39	0.12	0.07	0.07	0.07	0.39
	Absent*	0.00	0.08	0.41	0.12	0.08	0.08	0.08	0.41
	State Absent§	0.00	0.07	0.41	0.09	0.07	0.07	0.07	0.41
Non-Grain Agriculture/State emergence	None	0.04	0.04	0.37	0.04	0.04	0.04	0.37	0.37
	Absent*	0.04	0.04	0.40	0.04	0.04	0.04	0.39	0.39
Grain Agriculture/Taxation	None	0.00	0.09	0.09	0.09	0.09	0.09	0.09	0.09
	Absent*	0.00	0.18	0.18	0.17	0.18	0.18	0.17	0.17
	Tax Absent§	0.00	0.10	0.10	0.10	0.10	0.10	0.10	0.10
Taxation/Writing	None	0.00	0.05	0.05	0.06	0.06	0.06	0.06	0.05
	Absent*	0.00	0.11	0.12	0.12	0.12	0.12	0.12	0.08
Writing/State emergence	None	0.02	0.01	0.13	0.15	0.14	0.17	0.00	0.17
	Absent*	0.02	0.02	0.19	0.24	0.24	0.25	0.00	0.25

* Both traits absent at the ancestral node of the 10 largest global language families.

§ Grain unconstrained at the ancestral node of the 10 largest global language families.

Bibliography

1. Murdock GP. Ethnographic atlas - a summary. Ethnology. 1967;6(2).
2. Murdock GP, White DR. Standard cross-cultural sample. Ethnology. 1969;8(4):329-69.
3. Bouckaert R, Redding D, Sheehan O, Kyritsis T, Gray R, Jones KE, et al. Global language diversification is linked to socio-ecology and threat status. SocArXiv. 2022.
4. Ross MH. Political decision making and conflict: Additional cross-cultural codes and scales. Ethnology. 1983;22(2):169-92.
5. Murdock GP, Provost C. Measurement of Cultural Complexity. Ethnology. 1973;12(4):379-92.
6. Stasavage D. Biogeography, writing, and the origins of the state. In: Bisin A, Federico G, editors. The Handbook of Historical Economics: Academic Press; 2021. p. 881-902.
7. Basu S, Kirk M, Waymire G. Memory, transaction records, and The Wealth of Nations. Accounting, Organizations and Society. 2009;34(8):895-917.
8. Bianchini G, Sánchez-Baracaldo P. TreeViewer: Flexible, modular software to visualise and manipulate phylogenetic trees. Ecology and Evolution. 2024;14(2):e10873.
9. ArcGIS Pro 3.5.3 ed. p. Maps throughout this paper were created using ArcGIS® software by Esri.
ArcGIS® and ArcMap™ are the intellectual property of Esri and are used herein under license.
Copyright © Esri. All rights reserved. For more information about Esri® software, please visit www.esri.com.
10. Fritz SA, Purvis A. Selectivity in Mammalian Extinction Risk and Threat Types: a New Measure of Phylogenetic Signal Strength in Binary Traits. Conservation Biology. 2010;24(4):1042-51.
11. Pagel MD, Meade A. Bayesian analysis of correlated evolution of discrete characters by reversible-jump Markov chain Monte Carlo. American Naturalist. 2006;167(6):808-25.
12. Xie W, Lewis PO, Fan Y, Kuo L, Chen M-H. Improving Marginal Likelihood Estimation for Bayesian Phylogenetic Model Selection. Systematic Biology. 2011;60(2):150-60.
13. Kass RE, Raftery AE. Bayes factors. Journal of the American Statistical Association. 1995;90(430):773-95.
